# Epicatechin Isolated from *Litchi chinensis* Sonn. (Litchi) Fruit Peel Ethyl Acetate Extract Modulated Glucose Uptake in Chang Cells and Suppressed ROS Production in RAW 264.7 Macrophages

**DOI:** 10.3390/antiox13101233

**Published:** 2024-10-14

**Authors:** Gloria O. Izu, Nomonde P. Mapasa, Jennifer Nambooze, Maria S. Chukwuma, Emmanuel Mfotie Njoya, Gaetan T. Tabakam, Susanna L. Bonnet, Tshepiso J. Makhafola, Samson S. Mashele, Chika I. Chukwuma

**Affiliations:** 1 Department of Health Sciences, Faculty of Health and Environmental Sciences, Central University of Technology, Bloemfontein 9301, Free State, South Africa; 221039621@stud.cut.ac.za (G.O.I.); 224107123@cut.ac.za (N.P.M.); 2 Centre for Quality of Health and Living (CQHL), Faculty of Health and Environmental Sciences, Central University of Technology, Bloemfontein 9301, Free State, South Africa; enjoya@cut.ac.za (E.M.N.); tgaetan@cut.ac.za (G.T.T.); jmakhafola@cut.ac.za (T.J.M.); smashele@cut.ac.za (S.S.M.); 3 Department of Chemistry, Faculty of Natural and Agricultural Sciences, University of the Free State, Bloemfontein 9301, Free State, South Africa; 2020280521@ufs4life.ac.za (J.N.); 2017373891@ufs4life.ac.za (M.S.C.); bonnetsl@ufs.ac.za (S.L.B.)

**Keywords:** antioxidant, epicatechin, glucose uptake, isolation, litchi peel

## Abstract

Bioactive flavonoid epicatechin has been reported in the peel of litchi fruit but isolated from its hydroalcoholic extracts. This study isolated epicatechin with cellular glucose uptake modulatory and ROS production inhibitory properties from the ethyl acetate (EtOAc) extract using a bioassay-guided approach. The fruit peel was defatted with hexane and sequentially extracted using dichloromethane (DCM), EtOAc, methanol (MeOH) and water. In vitro phytochemical models, namely antioxidant (Fe^3+^ reducing, radical scavenging and anti-linoleic acid peroxidative) and glycaemic control (α-glucosidase and α-amylase inhibitory and glucose uptake modulatory), were employed for the bioassay-guided isolation, while the isolated compound was characterised using NMR and mass spectrometry and assessed for dose-dependent inhibition of α-glucosidase and lipopolysaccharide (LPS)-induced cellular ROS production, as well as modulation of cellular glucose uptake. Relative to the other extracts, the EtOAc extract had appreciable phenol and flavonoid contents, which perhaps influenced its potent anti-lipid peroxidative (65.0%) and α-glucosidase inhibitory (52.4%) effects. The α-glucosidase inhibitory potency of the fractions (1–8) from the EtOAc extracts correlated with their flavonoid contents, with fraction 5 outperforming other fractions. The fraction comprised a pool of fractions obtained from the DCM:MeOH:water (7:3:0.281 *v*/*v*/*v*) solvent system. LC-MS revealed the predominant presence of epicatechin in fraction 5, which was later isolated from one of the sub-fractions (sub-fraction 4) of fraction 5. This sub-fraction had stronger anti-lipid peroxidative (65.5%), α-glucosidase inhibitory (65.8%) and glucose uptake modulatory (38.2%) effects than the other sub-fractions from fraction 5, which could have been influenced by the isolated epicatechin. Moreover, the isolated epicatechin inhibited α-glucosidase (IC_50_ = 35.3 µM), modulated cellular glucose uptake (EC_50_ = 78.5 µM) and inhibited LPS-induced ROS production in RAW 264.7 macrophages in a dose-dependent fashion [IC_50_ = 18.9 µM; statistically comparable (*p* > 0.05) to ascorbic acid, IC_50_ = 9.57 µM]. Epicatechin from litchi peel EtOAc extract could potentiate glucose uptake modulatory, α-glucosidase inhibitory and ROS suppressive capacities, which could be influential in the use of litchi fruit peel for managing diabetes and associated oxidative damage.

## 1. Introduction

Diabetes remains a global health concern as it detrimentally affects the socio-economic sphere of many countries around the world. This has been aggravated by its ever-increasing prevalence, projected to increase from 537 million adults in 2021 to 783 million adults in 2045 [1]. The disease is classically characterised by high blood glucose caused by abnormal nutrient metabolism. Type 2 diabetes (T2D) remains the most prevalent type of diabetes [1], possibly because it is influenced by lifestyle-related factors, including dieting, weight gain and physical inactivity [2]. Insulin resistance is the main pathologic condition in T2D. Typically, it causes impaired metabolism of glucose. This includes drastically reduced uptake of circulating glucose into active cells of peripheral tissues due to the non-responsiveness of these cells to insulin signalling, which contributes to hyperglycaemia [3].

Hyperglycaemia-induced oxidative stress is pivotal in developing diabetic complications due to oxidative damages caused by reactive oxygen species (ROS) produced during persistent hyperglycaemia [4]. Notably, there is an elevated generation of superoxide radicals in respiring cells during hyperglycaemia [4]. Elevated radical production can trigger lipid peroxidation. ROS can oxidatively damage the lipid bilayer of cells through lipid peroxidation, comprising the integrity of the cells [4]. Moreover, lipid peroxidation processes can lead to the generation of more deleterious radical by-products, which can worsen oxidative stress. Furthermore, persistent hyperglycaemia can increase the rate of glycation of other biomolecules. This leads to the generation of advanced glycation end products (AGEs), biomarkers implicated in degenerative diseases like diabetes [4].

While there are many medications for managing diabetes and associated oxidative complications, dietary phytochemicals are increasingly becoming popular supplements to mitigate oxidative stress and manage metabolic diseases [5]. Many fruits are believed to contain antioxidant phenolics that influence their health benefits [6]. *Litchi chinensis* Sonn. (Sapindaceae) is a tropical and subtropical fruit with high commercial value due to its global consumption. It is known to have nutritional value and medicinal benefits [7]. Studies have shown that the fruit has antioxidant, anti-hyperglycaemic, anti-hypertensive, anti-obesogenic, anti-dyslipidaemic and hepatoprotective potentials [7]. Due to its commercial value, enormous amounts of by-products, including the peel and seeds, are generated from its processing, which contains bioactive phenolic compounds in their free and bound forms [8]. Thus, the fruit’s wastes could be of medicinal relevance.

In the context of antidiabetic potential, a previous review [9] has shown that more studies have been conducted on the seed of litchi fruit than the peel, warranting the need for more studies to probe the antidiabetic potential of the peel. The few antidiabetic studies that have been conducted suggest that fruit peel has glycaemic control potential. A study has shown that hydroalcoholic extracts of litchi peel have mild and strong inhibitory activity on α-amylase and α-glucosidase, respectively [10], suggesting its potential to suppress postprandial glycaemia. In another study, the peel flour and procyanidin-rich fraction from the peel reduced blood glucose and improved blood lipid profile in high-fat-diet-fed rats and mice [11,12]. A recent study further showed that litchi peel ethanol extract ameliorated T2D pathologies in db/db mice by reducing blood glucose, hepatic gluconeogenesis, oxidative stress and associated inflammatory responses [13]. Phytochemical assessments suggest that several key flavonoids, including catechins, procyanidins, proanthocyanidins, quercetin, rutin and their derivatives, may be influential in the antioxidant and other pharmacological properties of litchi peel [14,15,16].

The trend of previous diabetes-related pharmacological studies on litchi peel suggests that the focus has mostly been on the hydroalcoholic extracts, with limited studies on other extracts, such as the ethyl acetate extract [9]. Solvent extraction using ethyl acetate allows hydrogen bond formation with carboxyl and hydroxyl groups. It thus can result in the extraction of medium-polar to polar phenolics and/or flavonoids [17], which could influence potent medicinal properties. In fact, documented qualitative evidence has shown that litchi peel hydroalcoholic and ethyl acetate extracts have relatively similar proportions of phenolic compounds and flavonoids [18]. Concomitant pharmacological evidence in diabetic rats further suggests that the blood glucose lowering and antioxidant effect of litchi peel ethyl acetate extract outperforms that of the hydroalcoholic extract [18], suggesting that the ethyl acetate extract of litchi peel could also be a source of flavonoids with antioxidant and glycaemic control potential. However, this has not been explored and thus remains elusive. Previous studies reporting the isolation of some bioactive flavonoids (proanthocyanidin B4, proanthocyanidin B2 and epicatechin) from litchi peel were from the hydroalcoholic extract and not the ethyl acetate extract [14,15]. Thus, there is a need to explore litchi peel ethyl acetate extract as a source of key bioactive flavonoids. Therefore, in this study, we, for the first time, isolated a key bioactive flavonoid (epicatechin) from litchi peel ethyl acetate extract using an antioxidant and glycaemic control assay-guided approach, thus providing an alternative optimum approach to recovering bioactive epicatechin from litchi peel and more insight into the antidiabetic and antioxidant phytochemistry of litchi peel.

## 2. Materials Methods

### 2.1. Litchi Fruit Peel and Solvent Extraction

Litchi fruit (McLean’s Red cultivar) was purchased from a local fruit and vegetable store in Bloemfontein, South Africa. The fruit was washed with clean water. The peel was removed and oven-dried (Model No. 072160, Prolab instrument, Sep Sci., Johannesburg, South Africa) at 30 °C. The dried peel was pulverised. Approximately 300 g of the pulverised dried peel was defatted with 1.5 L of hexane and then sequentially extracted using 1.5 L of dichloromethane (DCM), ethyl acetate (EtOAc), methanol (MeOH) and water in order of increasing polarity. The extraction was done for 48 h at room temperature on an orbital shaker [OrbiShake, Model 262, Labotec 106 (Pty) Ltd., Johannesburg, South Africa] set at 125 rpm. The extraction was performed twice for each solvent. The DCM, EtOAc and MeOH extracts were concentrated using a Buchi 108 Rotavapor^®^ R-300 [Labotec (Pty) Ltd., Johannesburg, South Africa] and dried under a fume hood. The water extract was recovered by freeze drying (Martin Christ Alpha 1–2 LDplus Freeze Dryer 110, Separations, Johannesburg, South Africa). The extracts were weighed, transferred into clean screw-cap vials and stored at −20 °C.

### 2.2. Screening the Phenolic, Flavonoid, Antioxidant and Glycaemic Control Properties of Extracts

The extracts were screened at a concentration of 55 µg/mL. The total phenol and flavonoid contents were measured using previously described methods [19]. The total phenol and flavonoid contents were expressed in equivalents of gallic acid (mg/g GAE) and quercetin (mg/g QE), respectively.

For the antioxidant screening, the Fe^3+^ reducing (FRAP), 2,2-diphenyl-1-picrylhydrazyl (DPPH), 2,2′-azinobis-(3-ethylbenzothiazoline-6-sulfonic acid) (ABTS) and NO radicals scavenging and anti-linoleic acid peroxidative models were used, which were performed according to the protocols reported previously [19,20,21]. Ascorbic acid and Trolox were used as the positive control drugs.

For the glycaemic control screening, the extracts were tested for their ability to inhibit the activity of α-glucosidase and α-amylase, which was performed by adopting previously reported protocols [20]. Acarbose was used as the positive control drug.

### 2.3. Bioassay-Guided Fractionation of the Ethyl Acetate Extract and Isolation/Purification of Pure Compounds

**Figure 1:**
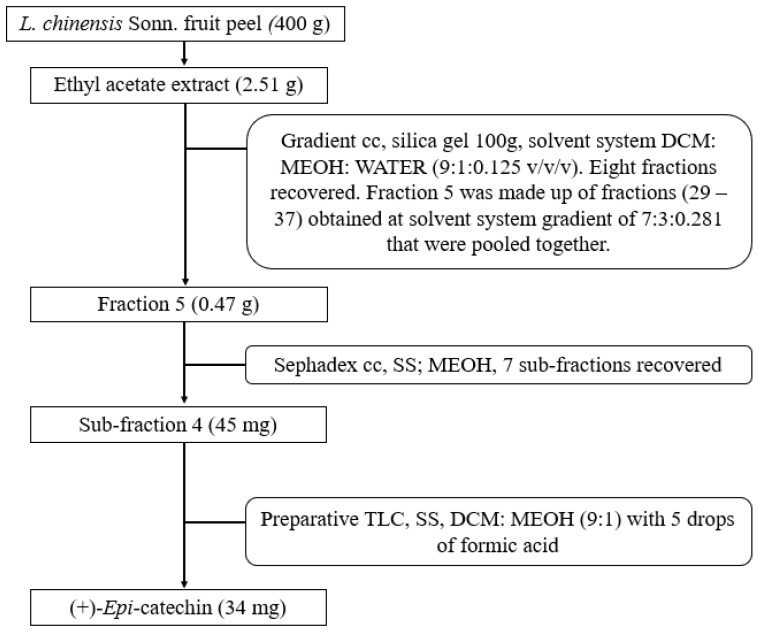
Schematic representation of the isolation of compounds from the ethyl acetate extract of litchi peel.

A flow diagram of the fractionation and isolation protocol is presented in Figure 1. The extract was subjected to a silica gel 60 (0.040–0.063 mm Merck) gradient column chromatography, starting with a solvent system (DCM:MeOH:water; 9:1:0.125 *v*/*v*/*v*), which was pre-determined by thin layer chromatography (TLC). The column was run with a decreasing volume of DCM, an increasing volume of MeOH and water, as shown in the Supplementary Materials (Table S1). The fractions obtained were pooled into 8 main fractions (Table S1) based on their TCL profiles. The fractions were identified as fractions 1 to 8.

The total phenol and flavonoid contents, as well as the antioxidant (FRAP, DPPH and ABTS radicals scavenging activity) and glycaemic control (α-glucosidase and α-amylase inhibition) potentials of the fractions were measured at a concentration of 35 µg/mL according to protocols previously reported [19,20].

Based on the bioactivity results obtained, fraction 5, which comprises a pool of fractions (29–37) obtained from a solvent system gradient of 7:3:0.281 *v*/*v*/*v* (DCM:MeOH:water), was selected for further fractionation using a Sephadex (Merck, Johannesburg, South Africa) column with 100% methanol as the solvent system. Also, LC-MS was used to chemically profile fraction 5 according to a protocol reported previously [22]. Using the accurate masses and extracted ions, the compounds in the fraction were tentatively determined and semi-quantified (mg/L) relative to catechin calibration and according to the extracted ions.

Seven sub-fractions were obtained from fraction 5 after separation in a Sephadex column. The in vitro radical (DPPH and ABTS) scavenging, anti-linoleic acid peroxidative, α-glucosidase inhibitory and cellular glucose uptake modulatory activities of the sub-fractions were measured at 20 µg/mL using methods reported in previous studies [20,21].

Chang cells were used to measure the cellular glucose uptake modulatory activity of the sub-fractions, and a previous protocol [20] was adopted after some modifications. Chang cells (ATCC^®^ CCL-13™; American Type Culture Collection Rockville, MD, USA) were cultured in a CO_2_ incubator using DMEM-high glucose media supplemented with 10% FBS (Separations, Johannesburg, South Africa). At confluence, the cells were seeded into a 48-well plate at a density of 15,000 cells per well. After 24 h of incubation, the spent media was replaced with 350 µL of fresh media containing the sub-fractions (20 µg/mL in incubation medium). After 24 h of incubation, the media was aspirated, and the cells were washed with PBS. Next, 350 µL of RPMI media (diluted to 8 mM glucose concentration) containing the sub-fractions (20 µg/mL in incubation medium) was added to the wells. Designated wells were used as the media (containing media without cells) and control (containing media with untreated cells) groups. The plate was incubated for 3 h. Thereafter, 20 µL aliquot from each well was diluted 24 times with distilled water and glucose concentration (in mg/mL) was measured in the diluted aliquots against a glucose standard curve (y = 9.766x + 0.0585) using a Glucose (GO) Assay Kit (Merck, Johannesburg, South Africa), which was then converted to concentration in millimolar (mM). The glucose uptake activity (%) of the control and test groups was computed relative to the media group using the following formula:

$$Glucose\;uptake\;activity\left(\%\right)=\frac{{GC}_{media}-{GC}_{control\;or\;test}}{{GC}_{media}}\times 100$$

where “GC” is the glucose concentration in millimolars (mMs).

The glucose uptake modulatory activity of the sub-fractions was computed relative to the control by subtracting the glucose uptake activity of the control from that of each fraction.

Based on the bioactivity data obtained from the subfractions, subfraction 4 was further subjected to preparative TLC (20 × 20 cm glass-backed, 0.25 mm silica plates) for the isolation of bioactive compound(s). The solvent system used was DCM: MEOH (9:1) with 5 drops of formic acid. The UV-active substances were scraped off, washed with acetone and methanol consecutively and recovered by filtration.

### 2.4. Nuclear Magnetic Resonance (NMR) Spectroscopy and Mass Spectrometry (MS)

The ^1^H and ^13^C NMR spectra were acquired using a Bruker Avance 400 MHz spectrometer. Deuterated DMSO solvent was used, and the working temperature was 25 °C. For ^1^H resonances (0 ppm), tetramethylsilane (TMS) was employed as an internal standard. The standard pulse sequences and processing macros given in the original program were used for advanced and two-dimensional spectra such as ^13^C APT/DEPT and 2D COSY, NOESY, HSQC and HMBC.

The compound(s) were subjected to low-resolution mass spectrometry. The MS experiment used an MD Sciex 3200 QTrap equipped with an electrospray (Turbo-ion spray) ionisation source.

### 2.5. Antioxidant and Glycaemic Control Examination of Isolated Compound

The isolated compound was assessed for dose-dependent antioxidant and glycaemic control potential using the following experimental models.

#### 2.5.1. Measuring the Inhibition of Lipopolysaccharide (LPS)-Induced ROS Production in RAW 264.7 Cells

The protocol reported in a recent study [22] was adopted for this assay after adding some modifications. RAW 264.7 macrophages [American Type Culture Collection (ATCC) Rockville, MD, USA); ATCC No.: TIB-71™] were cultured in a CO_2_ incubator (EC 160, Nϋve; Separations, Johannesburg, South Africa) using RPMI 1640 medium supplemented with 10% FBS under standard culture conditions. At appreciable confluence, 10,000 cells were seeded into the wells of a black/clear-bottom 96-well plate. The plate was incubated for 24 h before treating the cells with increasing concentrations (8.75, 17.5, 35, 70 and 140 µM) of the isolated compound and ascorbic acid (positive control). The treatment lasted for 2 h. Designated wells were used as the control and negative control groups, which were cells treated with water or DMSO (≤0.5%). After the 2 h treatment period elapsed, LPS (200 ng/mL in final incubation volume) was introduced into all the wells except those labelled as the control group. Rather, an equivalent volume of the LPS solvent was added to the control group wells. The plate was incubated for an additional 24 h. Thereafter, the cells were washed with PBS and exposed to 10 µM (dissolved in FBS-free culture medium) 2′,7′-dichlorodihydrofluorescein diacetate (DCFH-DA) (Merck, Johannesburg, South Africa) fluorescent probe for 30 min, which was performed in the dark. After the incubation period elapsed, the DCFH-DA-containing medium in the wells was replaced with PBS, and the fluorescence intensity of each well was measured at 485 nm (excitation) and 535 nm (emission) using a SpectraMax iD3 multi-mode microplate reader (Molecular Devices, San Jose, USA). Also, the images of the cells were visualised using a Leica DM IL LED inverted fluorescent microscope (Separations, Johannesburg, South Africa). The capacity of the compound or ascorbic acid to inhibit ROS production was computed as follows:

$$\mathrm{Inhibition}\;\left(\%\right)=\frac{\left(F_{\mathrm{Negative}\;\mathrm{control}}-F_{\mathrm{Normal}\;\mathrm{control}}\right)-(F_{\mathrm{Test}}-F_{\mathrm{Normal}\;\mathrm{control}})}{\left(F_{\mathrm{Negative}\;\mathrm{control}}-F_{\mathrm{Normal}\;\mathrm{control}}\right)}\;\times \;\frac{100}{1}$$

where “F” is the fluorescence intensity.

#### 2.5.2. Measuring the α-Glucosidase Inhibitory Potential of Isolated Compound

The dose-dependent α-glucosidase inhibitory potential of the isolated compound was measured according to a previously reported method [20] and was compared to that of acarbose. The isolated compound and acarbose were tested at the following concentrations: 3.75, 7.5, 15, 30 and 60 µM.

#### 2.5.3. Measuring the Glucose Uptake Activity of Isolated Compound in Chang Cells

The glucose uptake activity of the isolated compound was measured according to the glucose uptake protocol reported in Section 2.3 above. The glucose uptake activity of the isolated compound was tested at increasing concentrations (10, 20, 40 and 80 µM) and was compared to the activity of a 2 µM Metformin solution (positive control).

### 2.6. Data and Statistical Analysis

The data obtained were in triplicate analysis and were reported as the mean ± standard deviation. The IBM SPSS Statistics (Windows version 29.0) software was used to compute statistical significance (*p* < 0.05) when comparing the data (n = 3) between groups. The one-way ANOVA and Tukey post hoc were adopted for multiple comparative analyses.

The IC_50_ and EC_50_ values were computed from a non-linear fit of the transformed (Log10) tested concentrations versus the inhibitory or modulatory activities using GraphPad Prism 7 (Windows Version) software.

## 3. Results and Discussion

The antidiabetic and antioxidant potential of litchi peel has been documented [9,10,11,12,13]. However, these studies and the isolation of bioactive flavonoids (epicatechin and proanthocyanidins) from litchi peel have mostly been done on the hydroalcoholic extracts [9,10,11,12,13,14,15]. Documented evidence suggests that the ethyl acetate extract of litchi peel may possess stronger in vivo antidiabetic and antioxidant potential than the hydroalcoholic extract [18] and may be a source of the key bioactive flavonoids in litchi peel. However, the ethyl acetate extract of litchi peel has not been explored in this context. In this study we used bioassay-guided isolation to demonstrate for the first time that the ethyl acetate extract of litchi peel can be an alternative source to recover epicatechin with cellular glucose uptake modulatory and ROS production suppressive capacities.

### 3.1. Phytochemical and Bioactive Profiles of the Peel Extracts

Extraction of the peel with DCM, EtOAc, MEOH and water yielded 17.3 (5.74%), 2.51 (0.84%), 63.1 (21.0%) and 12.6 g (4.20%) of extracts, respectively. The EtOAc extract had the highest (*p* ˂ 0.05) total phenol content. In contrast, the MeOH and EtOAc extracts had significantly (*p* ˂ 0.05) higher total flavonoid contents than the DCM and water extracts (Table 1). The rich phenolic and flavonoid profile of the EtOAc extract could be linked to its ability to form hydrogen bonds with carboxyl and hydroxyl groups. It thus can extract medium polar to polar phenolics and/or flavonoids [17]. Perhaps the phenolic and flavonoid profile of the EtOAc extract also influenced its FRAP, radical scavenging and anti-linoleic acid peroxidative activities, which were stronger than those of the other extracts and statistically comparable (*p* > 0.05) to those of ascorbic acid and/or Trolox (Table 1). Phenolics have the ability to form stable phenoxy radicals, which influences their ability to scavenge radicals and chelate metals, making them good antioxidants [23,24]. Moreover, qualitative phytochemical analysis of litchi peel EtOAc extract and hydroalcoholic extracts has shown the presence of phenolic compounds and flavonoids in a relatively similar fashion and could be influential in the potent radical scavenging, FRAP and anti-lipid peroxidative activities demonstrated by the ethyl acetate and methanol extracts (Table 1) [14,15,16].

Compared to the other extracts, the EtOAc extract has the highest (*p* ˂ 0.05) α-glucosidase and α-amylase inhibitory activities (Table 1). The MeOH has moderate α-glucosidase inhibitory activity. The EtOAc and MeOH extracts show a stronger inhibitory activity on α-glucosidase than α-amylase, which is consistent with a recent report, where litchi peel hydroalcoholic extracts showed more potent inhibition on α-glucosidase than α-amylase [10]. The enzyme inhibitory effect of the polar extracts of litchi peel has been linked to the presence of notable flavonoids such as quercetin glycosides, proanthocyanidin, catechins and derivatives, etc. [10], which are also known to exert enzyme inhibitory action [25]. α-Glucosidase inhibitors exert glycaemic control by reducing postprandial blood glucose [26], reiterating the anti-hyperglycaemic potential of litchi peel.

### 3.2. Phytochemical and Bioactive Profiles of the Fractions and Sub-Fractions from the EtOAc Peel Extract

Gradient column chromatography yielded fractions that were pooled into eight major fractions (fractions 1–8) based on their TLC profiles (Table S2). Although fraction 5 has the lowest total phenol content, its flavonoid content is significantly (*p* ˂ 0.05) higher than other fractions (Table 2), which suggests that this fraction may contain some of the key bioactive flavonoids reported in litchi peel [10,14,15,16].

**Figure 2:**
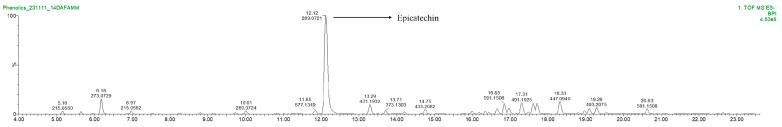
LC-MS chromatogram of fraction 5.

In fact, LC-MS analysis of fraction 5 showed the notable presence of epicatechin, among other flavonoids (Table 3 and Figure 2), which may have influenced its more robust (*p* ˂ 0.05) α-glucosidase and α-amylase inhibitory activity relative to the other fractions (Table 2). Moreover, the enzyme inhibitory activity of epicatechin and derivatives has been documented [27,28,29]. Fraction 5 also showed appreciable ABTS radical scavenging activity (Table 2) and thus was selected for further fractionation and isolation of bioactive flavonoids.

Subjecting fraction 5 to Sephadex chromatography yielded seven sub-fractions, which were assessed for their radical scavenging, anti-lipid peroxidative, α-glucosidase inhibitory and cellular glucose uptake activities. The anti-lipid peroxidative and α-glucosidase inhibitory activities of sub-fractions 4 and 5 significantly (*p* ˂ 0.05) outperform the activities of the other sub-fractions (Table 4). In fact, at a tested concentration of 20 µg/mL, their anti-lipid peroxidative and α-glucosidase activities were comparable to that of Trolox and acarbose, respectively. The data suggest that the epicatechin principle in fraction 5 (Table 3 and Figure 2) may have been eluted in sub-fractions 4 and 5, which influenced their potent activities. Moreover, the protective effect of epicatechin and derivatives on lipid peroxidative damage [30,31], as well as their α-glucosidase inhibitory effects [27,28,29], have been documented.

Furthermore, sub-fractions 4 and 5 show stronger (*p* ˂ 0.05) glucose uptake modulatory activities relative to the other sub-fractions in Chang cells (Table 4). However, sub-fraction 4 is not as potent as sub-fraction 5 (*p* ˂ 0.05). Cellular glucose uptake is a key mechanism in physiological glucose homeostasis, which is impaired during T2D due to insulin resistance [3]. Thus, the potent glucose uptake modulatory activity of sub-fraction 4 suggests it contains flavonoid(s) that can potentiate glycaemic control via improving insulin signalling and cellular glucose uptake and utilisation. Epicatechin was the most plausible influencing bioactive flavonoids of sub-fraction 4 because it was the most abundant flavonoid in the parent fraction (fraction 5) (Table 3 and Figure 2). It is a dietary supplement reported to improve cellular insulin sensitivity and glucose tolerance in obese and T2D individuals [32]. Hence, sub-fraction 4 was probed further to isolate bioactive epicatechin.

### 3.3. Spectrometric and Bioactive Profiles of Isolated Epicatechin

**Figure 3:**
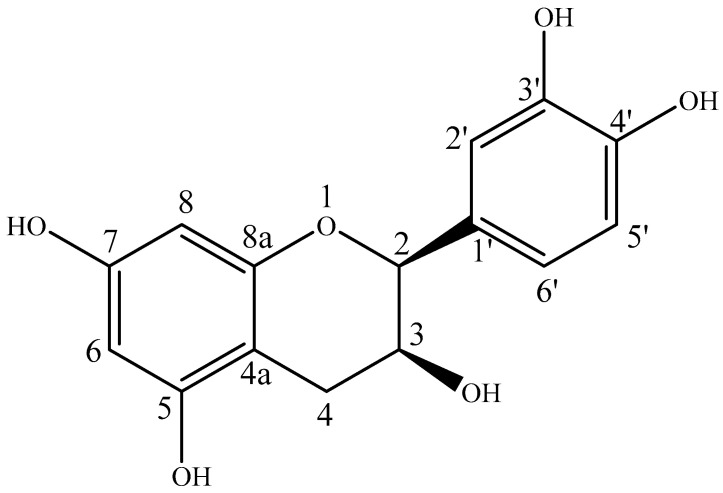
Structure of (+)-*Epi*-catechin.

Epicatechin was isolated from sub-fraction 4 using preparative TLC as a light/pale yellow powder. The spectral data of epicatechin (Figure 3) is as follows: ESI-MS^+^: *m/z* 291 [M + H]^+^, 313 [M + Na]^+^ and 387 [M + K]^+^ (Figure S1); ESI-MS^−^: *m/z* 289 [M − H]^−^, 325 [M + Cl]^−^ and 579 [2M − H]^−^ (Figure S2). ^1^H-NMR spectrum (400 MHz, CD_3_OD) is as shown in Table 5: δ_H_ 6.99 (1H, s, H-2′), δ_H_ 6.80 (1H, dd, *J* = 8.2 Hz, H-6′), δ_H_ 6.78 (1H, d, 8.1 Hz, H-5′), δ_H_ 5.96 (1H, d, H-6), δ_H_ 5.94 (1H, d, H-8), δ_H_ 4.84 (1H, brs, H-2), δ_H_ 4.19 (1H, s, H-3), δ_H_ 2.88 (1H, dd, *J* = 4.3, 16.8 Hz, H-4axi) and δ_H_ 2.75 (1H, dd, H-4eq), see Table 1. ^13^C-NMR (100 MHz, CD_3_OD): δ_C_ 156.6 (C-5), 156.3 (C-8a), 156.0 (C-7), 144.6 (C-3′), 144.4 (C-4′), 130.9 (C-1′), 113.9 (C-2′), 118.0 (C-6′), 114.5 (C-5′), 98.7 (C-4a), 95.0 (C-6), 94.5 (C-8), 78.5 (C-2), 66.1 (C-3) and 27.9 (C-4).

The ^1^H-NMR (400 MHz) spectrum of the compound showed four groups of chemical shift values attributable to the flavonoid’s skeleton [33]. The first group at δ_H_ 6.99 (1H, s, H-2′), δ_H_ 6.80 (1H, dd, *J* = 8.2 Hz, H-6′) and δ_H_ 6.78 (1H, d, 8.1 Hz, H-5′) form an ABX system (trisubstituted benzene ring-B of flavonoids); the second group at δ_H_ 5.96 (1H, d, H-6) and δ_H_ 5.94 (1H, d, H-8) correspond to an AX system (tetrasubstituted benzene ring-A of flavonoids); the third and fourth groups formed by two oxymethine protons at δ_H_ 4.84 (1H, brs, H-2) and δ_H_ 4.19 (1H, s, H-3); and two methylene anisotropic protons at δ_H_ 2.88 (1H, dd, *J* = 4.3, 16.8 Hz, H-4axi) and δ_H_ 2.75 (1H, dd, H-4eq) (Figure S3) are attributable to proton 2, 3 and 4 of saturated ring C of flavan-3-ol type flavonoid, respectively [33,34]. The ^13^C-NMR (100 MHz, CD_3_OD) spectrum indicated the presence of 15 carbon atoms [five oxygenated aromatic carbons at δ_C_ 156.6 (C-5), 156.3 (C-8a), 156.0 (C-7), 144.6 (C-3′) and 144.4 (C-4′); five aromatic methine carbons at 113.9 (C-2′), 118.0 (C-6′), 114.5 (C-5′), 95.0 (C-6) and 94.5 (C-8); two aromatic quaternary carbons at 130.9 (C-1′) and 98.7 (C-4a); two aliphatic oxygenated methine carbons at 78.5 (C-2) and 66.1 (C-3), and one aliphatic methylene carbon at 27.9 (C-4)] (Figure S4). The DEPT 135 spectrum confirms the presence of one methylene and seven methine carbons. All the above above-mentioned information confirms compound 1 to be a flavan-3-ol flavonoid type [35]. In the HSQC, HMBC, COSY and NOESY spectra (Figures S5–S8), the structure of the compound was a catechin epimer. The presence of signals of asymmetric carbons around δ 79 (C-2) and 67 (C-3) shows the compound is an epicatechin rather than a catechin [signals around δ 82 (C-2) and 68 (C-3)]. The chemical shift values of protons H-2 and H-3 around δ 4.85 and 4.18 confirms the compound is a (+)-*Epi*-catechin [36], which has been previously isolated from *Euphorbia thymifolia* linn. and characterised [37], as shown in Table 5.

**Table 5:** ^1^H (400 MHz) and ^13^C (100 MHz) NMR data of the isolated compound compared with reported literature.

Position	Compound 1		Reported Literature [36]	
	δ ^1^H (*J* in Hz)	δ ^13^C	δ ^1^H (*J* in Hz)	δ ^13^C
**2**	4.84 (s)	78.5	4.85 (s)	79.6
**3**	4.19 (s)	66.1	4.18 (br s)	67.1
**4axi**	2.88 (dd, *J* = 4.3; 16.8 Hz)	27.9	2.83 (br d, 17.0 Hz)	29.2
**4eq**	2.75 (dd, *J* = 4.3; 16.8 Hz)	27.9	2.70 (br d, 17.0 Hz)	29.2
**4a**		98.7		100.0
**5**		156.6		157.8
**6**	5.96 (d)	95.0	5.99 (d, *J* = 1.8 Hz)	96.3
**7**	5.94 (d)	156.0	5.88 (d, *J* = 1.8 Hz)	157.8
**8**		94.5		95.5
8a		156.3		157.4
1′		130.9		132.5
2′	6.99 (s)	113.9	7.01 (d, 1.8 Hz)	115.5
3′		144.6		145.6
4′		144.4		145.5
5′	6.78 (d, *J* = 8.1 Hz)	114.5	6.75 (d, *J* = 8.1 Hz)	115.7
6′	6.80 (dd, *J* = 8.1; 2.2 Hz)	118.0	6.80 (dd, *J* = 8.0; 1.8 Hz)	119.6

**Figure 4:**
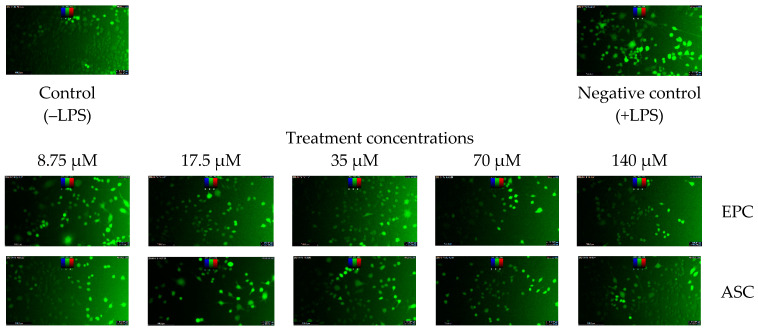

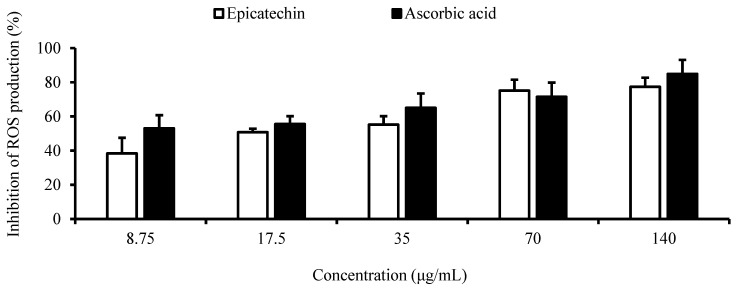
Dose-dependent inhibitory effect of epicatechin (EPC) and ascorbic acid (ASC) on ROS production in LPS-treated RAW 264.7 macrophages. Data are presented as mean ± SD of triplicate analysis. The letters at the top of the bars represent significant differences (*p* < 0.05) between groups at a given concentration when there are no similar letters. “LPS” means lipopolysaccharide.

Bioactivity assessment of the isolated epicatechin shows that it demonstrates a dose-dependent inhibitory action (IC_50_ = 18.9 µM) on LPS-induced ROS production in RAW 264.7 macrophages, which is statistically comparable (*p* > 0.05) to that of ascorbic acid (IC_50_ = 9.57 µM) (Table 6 and Figure 4), reiterating the antioxidant potency of epicatechin. LPS-induced ROS production has been linked to proinflammatory signalling, which can disrupt cellular processes and cause oxidative tissue injuries due to the excessive production of superoxide ions and hydrogen peroxide [38]. Hence, it is plausible that the inhibitory effect of epicatechin on LPS-induced ROS production in RAW 264.7 macrophages was linked to both antioxidant and anti-inflammatory capacities. Moreover, the inhibitory effect of epicatechin on LPS-induced production of proinflammatory mediators and cytokines in RAW 264.7 macrophages has been reported [39].

**Figure 5:**
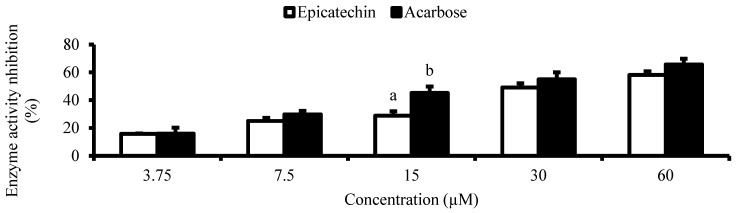
Dose-dependent inhibitory effect of epicatechin and acarbose on α-glucosidase activity. Data are presented as mean ± SD of triplicate analysis. The letters at the top of the bars represent significant differences (*p* < 0.05) between groups at a given concentration when there are no similar letters.

**Figure 6:**
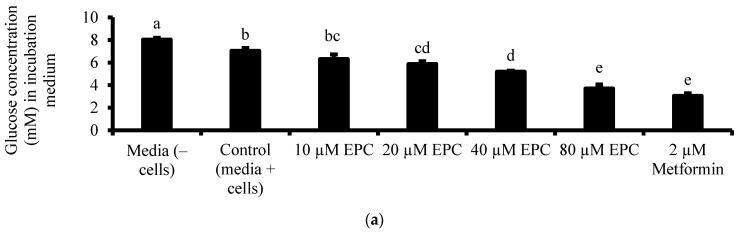

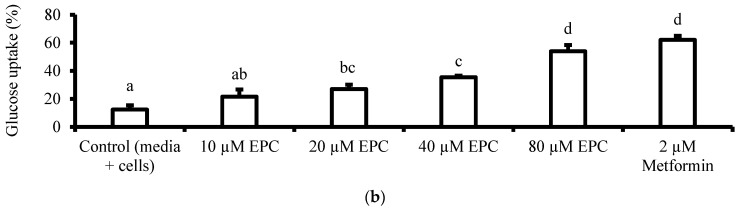
(**a**) Glucose concentration in the medium of cells receiving different treatments and (**b**) % glucose uptake of cells receiving different treatments. Data are presented as mean ± SD of triplicate analysis. The letters at the top of the bars represent significant differences (*p* < 0.05) between the treatments when there are no similar letters. “EPC” means epicatechin.

Furthermore, the isolated epicatechin demonstrated a dose-dependent glycaemic control potential. It has an inhibitory effect (IC_50_ = 35.3 µM) on α-glucosidase (Table 6 and Figure 5), suggesting it could be a key flavonoid influencing the α-glucosidase inhibitory activity of the potent sub-fraction(s)/fraction(s) and extract of litchi fruit peel. In Chang cells, epicatechin exerts a dose-dependent modulatory effect (EC_50_ = 78.5 µM) on glucose uptake (Table 6 and Figure 6).

Relative to the untreated cells (control), an 80 µM epicatechin concentration enhances (*p* ˂ 0.05) glucose uptake to an extent that is statistically comparable (*p* > 0.05) to the glucose uptake effect of a 2 µM Metformin, suggesting epicatechin may be effective in promoting cellular glucose uptake in cells of peripheral tissues, and may have influenced the glucose uptake activity of the potent sub-fractions 4 and 5 (Table 4). Documented evidence suggests that epicatechin may enhance glucose uptake by modulating insulin signalling via the PI3K/AKT signalling pathway and modulating the translocation of glucose transporters [28,40]. Further studies are, however, needed to investigate this speculation.

## 4. Conclusions

In the present study, a bioassay-guided approach was used to probe into litchi fruit peel’s antioxidant and anti-hyperglycaemic phytochemistry. Ethyl acetate solvent effectively extracted key flavonoids, which potentiated more potent antioxidant and enzyme inhibitory activities relative to the other solvent extraction. The flavonoid contents of fractions from the ethyl acetate extract also correlated with their α-glucosidase inhibitory activities. Epicatechin was the most abundant flavonoid in the fraction (fraction 5), with the highest inhibitory activity. Epicatechin was isolated from a sub-fraction (sub-fraction 4) of fraction 5. The isolated epicatechin demonstrated a dose-dependent inhibitory effect on α-glucosidase, modulatory effect on cellular glucose uptake, inhibitory effect on cellular ROS production, and possibly influenced the potent α-glucosidase inhibitory, glucose uptake modulatory and anti-lipid peroxidative activity demonstrated by sub-fraction 4. The data of this study showed that litchi peel EtOAc extract may exert antioxidant and glycaemic control by alleviating ROS-mediated oxidative insults, inhibiting carbohydrate digesting enzymes and modulating cell glucose uptake, and epicatechin may be a principal bioactive flavonoid influencing these antioxidant and glycaemic control properties. Thus, litchi peel EtOAc extract can be an alternative source to recover epicatechin with cellular glucose uptake modulatory and ROS production suppressive capacities.

## Figures and Tables

**Table 1:** Total phenol and flavonoid contents and in vitro Fe^3+^ reducing, radical scavenging anti-linoleic acid peroxidative and enzyme inhibitory activities of crude extracts at 55 µg/mL.

Extracts and Standards	TPC (mg/g GAE)	TFC (mg/g QE)	FRAP (mg/g GAE)	DPPH (%)	ABTS (%)	NO (%)	AnLP (%)	AGI (%)	AAI (%)
DCM	39.7 ± 1.35 ^a^	35.3 ± 9.71 ^a^	151 ± 5.77 ^a^	46.1 ± 5.07 ^a^	43.6 ± 0.43 ^b^	5.63 ± 2.77 ^a^	33.6 ± 6.28 ^a^	5.10 ± 3.54 ^a^	9.84 ± 4.64 ^a^
EtOAc	435 ± 22.4 ^c^	109.5 ± 12.1 ^c^	600 ± 54.7 ^c^	83.3 ± 2.01 ^b^	96.6 ± 0.26 ^d^	15.0 ± 5.22 ^b^	65.0 ± 3.48 ^b^	52.4 ± 4.63 ^d^	33.7 ± 7.42 ^bc^
MeOH	152 ± 2.41 ^b^	126 ± 13.0 ^c^	381 ± 10.5 ^b^	44.8 ± 8.55 ^a^	81.9 ± 0.89 ^c^	10.0 ± 0.68 ^ab^	35.9 ± 3.68 ^a^	34.1 ± 3.14 ^c^	13.8 ± 5.16 ^a^
Water	55.9 ± 1.75 ^a^	68.9 ± 3.32 ^b^	179 ± 11.9 ^a^	36.1 ± 1.56 ^a^	23.1 ± 1.10 ^a^	22.2 ± 1.21 ^c^	28.0 ± 4.65 ^a^	17.6 ± 5.18 ^b^	25.7 ± 4.09 ^ab^
Asc	N/A	N/A	879 ± 12.0 ^d^	45.3 ± 5.03 ^a^	97.9 ± 0.06 ^de^	4.41 ± 1.82 ^a^	ND	N/A	N/A
Trolox	N/A	N/A	657 ± 16.9 ^c^	91.9 ± 3.49 ^b^	99.3 ± 1.10 ^e^	13.5 ± 2.05 ^b^	84.1 ± 5.12 ^c^	N/A	N/A
Aca	N/A	N/A	N/A	N/A	N/A	N/A	N/A	88.3 ± 1.31 ^e^	43.6 ± 11.1 ^c^

“AAI”, α-amylase inhibitory activity; “ABTS”, 2,2′-azino-bis(3-ethylbenzothiazoline-6-sulfonic acid) radical scavenging activity; “Aca”, acarbose; “AGI”, α-glucosidase inhibitory activity; “AnLP”, anti-linoleic acid peroxidation activity; “Asc”, ascorbic acid; “DCM”, dichloromethane extract; “DPPH”, 2,2-diphenyl-1-picrylhydrazyl radical scavenging activity; “FRAP”, Fe^3+^ reducing antioxidant power; “EtOAc”, ethyl acetate extract; “GAE”, gallic acid equivalent; “MeOH”, methanol extract; “N/A”, not applicable; “ND”, not determined; “NO”, nitric oxide radical scavenging activity; “QE”, quercetin equivalent; “TFC”, total flavonoid content; “TPC”, total phenol content. Data are presented as mean ± SD of triplicate analysis. For each assay, the superscript letters represent significant differences (*p* < 0.05) between groups when there are no similar letters.

**Table 2:** Total phenol and flavonoid contents and in vitro Fe^3+^ reducing, radical scavenging and enzyme inhibitory activities of fractions from the crude ethyl acetate extract at 35 µg/mL.

Fractions and Standards	TPC (mg/g GAE)	TFC (mg/g QE)	FRAP (mg/g GAE)	DPPH (%)	ABTS (%)	AGI (%)	AAI (%)
F1	361 ± 66.0 ^de^	42.2 ± 8.43 ^b^	167 ± 3.08 ^d^	40.7 ± 6.98 ^bc^	80.6 ± 1.42 ^a^	22.0 ± 4.44 ^a^	14.1 ± 4.98 ^ab^
F2	322 ± 55.2 ^d^	71.7 ± 5.65 ^c^	166 ± 3.12 ^d^	34.7 ± 4.67 ^bc^	80.1 ± 1.73 ^a^	37.7 ± 5.31 ^bc^	15.1 ± 3.19 ^ab^
F3	220 ± 19.5 ^bc^	73.5 ± 8.55 ^c^	124 ± 16.5 ^b^	14.9 ± 1.30 ^a^	77.9 ± 6.00 ^a^	49.3 ± 8.13 ^cd^	22.9 ± 9.33 ^b^
F4	195 ± 19.7 ^ab^	70.8 ± 16.0 ^c^	78.3 ± 12.2 ^a^	27.8 ± 6.27 ^ab^	97.6 ± 2.22 ^d^	37.5 ± 5.98 ^bc^	7.20 ± 3.57 ^a^
F5	133 ± 25.8 ^a^	104 ± 6.11 ^d^	122 ± 6.70 ^b^	28.3 ± 5.14 ^b^	98.2 ± 1.17 ^d^	68.1 ± 1.51 ^e^	36.7 ± 4.86 ^c^
F6	287 ± 4.33 ^cd^	54.1 ± 7.50 ^bc^	166 ± 1.36 ^d^	39.4 ± 8.08 ^bc^	98.0 ± 0.06 ^d^	31.7 ± 7.40 ^ab^	6.39 ± 1.82 ^a^
F7	422 ± 3.88 ^e^	18.9 ± 2.93 ^a^	179 ± 2.05 ^de^	43.1 ± 1.05 ^c^	97.4 ± 2.21 ^d^	18.4 ± 1.87 ^a^	14.6 ± 2.30 ^ab^
F8	186 ± 16.4 ^ab^	18.2 ± 2.12 ^a^	145 ± 4.87 ^c^	38.2 ± 0.73 ^bc^	96.9 ± 1.08 ^cd^	21.9 ± 5.52 ^a^	10.8 ± 3.74 ^ab^
Asc	N/A	N/A	290 ± 4.36 ^f^	41.4 ± 1.89 ^c^	90.3 ± 1.88 ^bc^	N/A	N/A
Trolox	N/A	N/A	192 ± 3.93 ^e^	79.0 ± 4.13 ^d^	87.0 ± 1.50 ^ab^	N/A	N/A
Aca	N/A	N/A	N/A	N/A	N/A	65.8 ± 2.76 ^e^	39.9 ± 3.45 ^c^

“AAI”, α-amylase inhibitory activity; “ABTS”, 2,2′-azino-bis(3-ethylbenzothiazoline-6-sulfonic acid) radical scavenging activity; “Aca”, acarbose; “AGI”, α-glucosidase inhibitory activity; “Asc”, ascorbic acid; “DPPH”, 2,2-diphenyl-1-picrylhydrazyl radical scavenging activity; “FRAP”, Fe^3+^ reducing antioxidant power; “GAE”, gallic acid equivalent; “N/A”, not applicable; “QE”, quercetin equivalent; “TFC”, total flavonoid content; “TPC”, total phenol content. Data are presented as mean ± SD of triplicate analysis. For each assay, the superscript letters represent significant differences (*p* < 0.05) between groups when there are no similar letters.

**Table 3:** LC-MS data of fraction 5.

Identified Compounds	Ontology	ART (min)	AMz	Total Score	Peak Height	Conc. (mg/L)
Procyanidin B5	Biflavonoids and polyflavonoids	8.241	577.13507	7.6791	1280	0.3
Pseudolaroside B;(-)-Pseudolaroside B	Hydrolysable tannins	8.802	329.0878	7.4069	2855	0.7
Butyl 3-O-caffeoylquinate	Quinic acids and derivatives	9.147	409.15076	7.3007	3121	0.8
Glucosyringic acid;Syringin 4-O-beta-glucoside	Hydrolysable tannins	9.722	359.09879	7.349	5648	1.4
Epicatechin	Catechins	12.133	289.07214	8.0017	370,407	94.5
Kievitone hydrate	8-prenylated isoflavanones	13.707	373.13028	4.7465	14,903	3.8
Garcimangosone D	Phenolic glycosides	14.2	391.1037	7.3375	4686	1.2
5-hydroxy-2-(4-hydroxyphenyl)-7-{[3,4,5-trihydroxy-6-(hydroxymethyl)oxan-2-yl]oxy}-3,4-dihydro-2H-1-benzopyran-4-one	Flavonoid-7-O-glycosides	15.163	433.11365	7.5754	1137	0.3
Garcimangosone D	Phenolic glycosides	15.254	391.1037	7.0551	1280	0.3
Polydine	Flavonoid-7-O-glycosides	16.175	421.11395	7.3603	4486	1.1
Isolariciresinol 9’-O-beta-D-glucoside	Lignan glycosides	16.435	521.20264	7.1728	7835	2.0
Quercetin 3-galactoside	Flavonoid-3-O-glycosides	16.659	463.0878	7.7967	16,029	4.1
Aspalathin	2’-Hydroxy-dihydrochalcones	16.834	451.12402	7.1687	7039	1.8
Aurasperone C	Naphthopyranones	16.843	591.15076	7.5058	27,229	6.9
Isolariciresinol 9’-O-beta-D-glucoside	Lignan glycosides	16.97	521.20251	6.0419	25,518	6.5
Aurovertin G	C-glycosyl compounds	17.308	491.19247	6.3281	50,720	12.9
Hovenitin I	Epigallocatechins	17.492	333.06146	8.0014	9169	2.3
Avicularin	Flavonoid-3-O-glycosides	17.611	433.07831	6.7129	46,840	11.9
Quercitrin	Flavonoid-3-O-glycosides	18.322	447.09387	7.9544	46,688	11.9
Ethyl 7-epi-12-hydroxyjasmonate glucoside	Fatty acyl glycosides of mono- and disaccharides	18.513	415.19803	6.1113	294	0.1
Kaempferol 3-alpha-L-arabinopyranoside	Flavonoid glycoside	18.969	417.08301	7.5182	11,122	2.8
Phlorizin	Polyphenol glycoside	19.097	435.1297	7.9116	24,326	6.2

“ART”, average retention time”; “AMz”, average mass-to-charge ratio; “Conc.”, concentration.

**Table 4:** In vitro radical scavenging, anti-linoleic acid peroxidative, enzyme inhibitory and glucose uptake modulatory activities of sub-fractions at 20 µg/mL.

Sub-Fractions and Standards	DPPH (%)	ABTS (%)	AnLP (%)	AGI (%)	GUMA (%)
SBF1	37.7 ± 4.26 ^cd^	84.8 ± 3.96 ^bc^	25.9 ± 7.00 ^ab^	22.4 ± 2.69 ^a^	NAD
SBF2	26.7 ± 6.24 ^abc^	72.8 ± 1.34 ^a^	19.9 ± 2.21 ^a^	36.4 ± 4.03 ^ab^	NAD
SBF3	22.1 ± 3.81 ^ab^	72.1 ± 4.63 ^a^	38.7 ± 5.52 ^b^	46.8 ± 12.7 ^bc^	14.0 ± 6.92 ^a^
SBF4	32.2 ± 6.36 ^bcd^	86.8 ± 2.54 ^c^	65.5 ± 8.95 ^c^	65.8 ± 2.85 ^d^	38.2 ± 5.81 ^c^
SBF5	26.1 ± 1.99 ^abc^	87.1 ± 0.90 ^c^	60.1 ± 3.72 ^c^	56.0 ± 3.00 ^cd^	28.0 ± 2.47 ^b^
SBF6	18.2 ± 4.00 ^a^	91.8 ± 4.31 ^c^	29.6 ± 7.80 ^ab^	29.2 ± 6.00 ^a^	NAD
SBF7	15.3 ± 4.89 ^a^	86.0 ± 1.71 ^c^	23.4 ± 6.95 ^ab^	23.9 ± 5.22 ^a^	11.7 ± 3.28 ^a^
Asc	40.0 ± 2.06 ^d^	75.9 ± 2.77 ^ab^	ND	N/A	N/A
Trolox	68.2 ± 3.87 ^e^	76.6 ± 1.83 ^ab^	70.7 ± 4.05 ^c^	N/A	N/A
Aca	N/A	N/A	N/A	58.8 ± 4.04 ^cd^	N/A

“ABTS”, 2,2′-azino-bis(3-ethylbenzothiazoline-6-sulfonic acid) radical scavenging activity; “Aca”, acarbose; “AGI”, α-glucosidase inhibitory activity; “AnLP”, anti-linoleic acid peroxidation activity; “Asc”, ascorbic acid; “DPPH”, 2,2-diphenyl-1-picrylhydrazyl radical scavenging activity; “GUMA”, glucose uptake modulatory activity; “N/A”, not applicable; “ND”, not determined. Data are presented as mean ± SD of triplicate analysis. For each assay, the superscript letters represent significant differences (*p* < 0.05) between groups when there are no similar letters.

**Table 6:** IC_50_ and EC_50_ values for the dose-dependent activities of the tested samples.

Assays	Epicatechin	Acarbose	Ascorbic Acid
	IC_50_ and EC_50_ Values (µM)		
α-Glucosidase inhibitory activity (IC_50_)	35.3 ± 5.75 ^a^	11.0 ± 4.11 ^b^	NA
Inhibition of ROS production in RAW 264.7 cells (IC_50_)	18.9 ± 5.44	NA	9.57 ± 4.10
Chang cell glucose uptake modulatory activity (EC_50_)	78.5 ± 13.6	NA	NA

“NA” means “not applicable”. Data are presented as mean ± SD of triplicate analysis. For each assay, the superscript letters attached to each dataset represent significant differences (*p* ˂ 0.05) between groups when there are no similar letters.

## Data Availability

The data of this study are available from the corresponding author upon request.

## Supplementary Materials

The following supporting information can be downloaded at: https://www.mdpi.com/article/10.3390/antiox13101233/s1, Figure S1: ESIMS (+) spectrum of the isolated compound; Figure S2: ESIMS (-) spectrum of the isolated compound; Figure S3: ^1^H NMR (400 MHz, CD_3_OD) spectrum of the isolated compound; Figure S4: ^13^C NMR (100 MHz, CD_3_OD) spectrum of the isolated compound; Figure S5: HSQC spectrum (CD_3_OD) of the isolated compound; Figure S6: HMBC spectrum (CD_3_OD) of the isolated compound; Figure S7: COSY spectrum (CD_3_OD) of the isolated compound; Figure S8: NOESY spectrum (CD_3_OD) of the isolated compound; Table S1: Non-polar to polar eluent system used gradient column fractionation of the EtAc extract; Table S2: Yield of fractions from gradient column chromatography of the EtAc extract.

## References

[1] International Diabetes Federation (2021). IDF Diabetes Atlas.

[2] Zhu J., Chen M., Pang Y., Li S. (2021). Impact of lifestyle education for type 2 diabetes mellitus: Protocol for a randomized controlled trial. J. Med..

[3] Galicia-Garcia U., Benito-Vicente A., Jebari S., Larrea-Sebal A., Siddiqi H., Uribe K.B., Ostolaza H., Martín C. (2020). Pathophysiology of Type 2 Diabetes Mellitus. Int. J. Mol. Sci..

[4] Bhatti J.S., Sehrawat A., Mishra J., Sidhu I.S., Navik U., Khullar N., Kumar S., Bhatti G.K., Reddy P.H. (2022). Oxidative stress in the pathophysiology of type 2 diabetes and related complications: Current therapeutics strategies and future perspectives. Free Radic. Biol. Med..

[5] Sun C., Zhao C., Guven E., Paoli P., Simal-Gándara J., Ramkumar K.M., Wang S., Buleu F.N., Pah A.M., Turi V. (2020). Dietary polyphenols as antidiabetic agents: Advances and opportunities. Food Front..

[6] Afam I.O.J., Silungwe H., Takalani T., Omolola A.O., Udeh H.O., Anyasi T.A. (2021). Antioxidant-rich natural fruit and vegetable products and human health. Int. J. Food Prop..

[7] Zhao L., Wang K., Wang K., Zhu J., Hu Z. (2020). Nutrient components, health benefits, and safety of litchi (*Litchi chinensis* Sonn.): A review. Compr. Rev. Food Sci. Food Saf..

[8] Cano-Gómez C.I., Alonso-Castro A.J., Carranza-Alvarez C., Wong-Paz J.E. (2024). Advancements in *Litchi chinensis* Peel Processing: A Scientific Review of Drying, Extraction, and Isolation of Its Bioactive Compounds. Foods.

[9] Chukwuma C.I., Izu G.O., Chukwuma M.S., Samson M.S., Makhafola T.J., Erukainure O.L. (2021). A review on the medicinal potential, toxicology, and phytochemistry of litchi fruit peel and seed. J. Food Biochem..

[10] Tanaka M.R.R., Mataruco L.d.S., Frota E.G., Coelho B.E.S., Lima M.d.S., Pimentel T.C., Barão C.E. (2024). Lychee peel extract obtained by ultrasound-assisted extraction: Bioactive compounds and functional properties. Acta Sci. Technol..

[11] Rong S., Hu X., Zhao S., Zhao Y., Xiao X., Bao W., Liu L. (2017). Procyanidins extracted from the litchi pericarp ameliorate atherosclerosis in ApoE knockout mice: Their effects on nitric oxide bioavailability and oxidative stress. Food Funct..

[12] Queiroz E.R., Abreu C., Rocha D.A., Sousa R.V., Fráguas R.M., Braga M.A., César P. (2018). Lychee (*Litchi chinensis* Sonn.) peel flour: Effects on hepatoprotection and dyslipidemia induced by a hypercholesterolemic diet. An. Acad. Bras. Ciênc..

[13] Yang Z., Zhang L., Liu J., Li D. (2024). Litchi Pericarp Extract Treats Type 2 Diabetes Mellitus by Regulating Oxidative Stress, Inflammatory Response, and Energy Metabolism. Antioxidants..

[14] Zhao M., Yang B., Wang J., Li B., Jiang Y. (2006). Identification of the major flavonoids from pericarp tissues of lychee fruit in relation to their antioxidant activities. Food Chem..

[15] Zhao M., Yang B., Wang J., Liu Y., Yu L., Jiang Y. (2007). Immunomodulatory and anticancer activities of flavonoids extracted from litchi (*Litchi chinensis* Sonn) pericarp. Int. Immunopharmacol..

[16] Li J., Jiang Y. (2007). Litchi Flavonoids: Isolation, Identification and Biological Activity. Molecules.

[17] Prasad K.N., Chew L.Y., Khoo H.E., Kong K.W., Azlan A., Ismail A. (2010). Antioxidant capacities of peel, pulp, and seed fractions of *Canarium odontophyllum* Miq. fruit. J. Biomed. Biotechnol..

[18] Chauhan S., Gupta S., Yasmin S., Saini M. (2021). Antihyperglycemic and Antioxidant Potential of Plant Extract of *Litchi chinensis* and *Glycine max*. Int. J. Nutr. Pharmacol. Neurol. Dis..

[19] Chukwuma C.I., Mashele S.S., Akuru E.A. (2020). Evaluation of the in vitro ⍺-amylase inhibitory, antiglycation, and antioxidant properties of *Punica granatum* L. (pomegranate) fruit peel acetone extract and its effect on glucose uptake and oxidative stress in hepatocytes. Food Biochem..

[20] Motloung D.M., Mashele S.S., Matowane G.R., Swain S.S., Bonnet S.L., Noreljaleel A.E.M., Oyedemi S.O., Chukwuma C.I. (2020). Synthesis, characterization, antidiabetic and antioxidative evaluation of a novel Zn(II)-gallic acid complex with multi-facet activity. J. Pharm. Pharmacol..

[21] Mashile B., Setlhodi R., Izu G.O., Erukainure O.L., Mashele S.S., Makhafola T.J., Eze K.C., Chukwuma C.I. (2024). Temperature-dependent extraction and chromatographic recovery and characterization of ellagitannins with potent antioxidant and glycaemic control properties from “Wonderful” pomegranate peel. Int. J. Food Sci. Technol..

[22] Izu G.O., Mfotie Njoya E., Tabakam G.T., Nambooze J., Otukile K.P., Tsoeu S.E., Fasiku V.O., Adegoke A.M., Erukainure O.L., Mashele S.S. (2024). Unravelling the Influence of Chlorogenic Acid on the Antioxidant Phytochemistry of Avocado (*Persea americana* Mill.) Fruit Peel. Antioxidants.

[23] Chen J., Yang J., Ma L., Li J., Shahzad N., Kim C.K. (2020). Structure-antioxidant activity relationship of methoxy, phenolic hydroxyl, and carboxylic acid groups of phenolic acids. Sci. Rep..

[24] Scarano A., Laddomada B., Blando F., De Santis S., Verna G., Chieppa M., Santino A. (2023). The Chelating Ability of Plant Polyphenols Can Affect Iron Homeostasis and Gut Microbiota. Antioxidants.

[25] Dirir A.M., Daou M., Yousef A.F., Yousef L.F. (2022). A review of alpha-glucosidase inhibitors from plants as potential candidates for the treatment of type-2 diabetes. Phytochem. Rev. Proc. Phytochem. Lett..

[26] Kashtoh H., Baek K.-H. (2022). Recent Updates on Phytoconstituent Alpha-Glucosidase Inhibitors: An Approach towards the Treatment of Type Two Diabetes. Plants.

[27] Wu X., Hu M., Hu X., Ding H., Gong D., Zhang G. (2019). Inhibitory mechanism of epicatechin gallate on α-amylase and α-glucosidase and its combinational effect with acarbose or epigallocatechin gallate. J. Mol. Liq..

[28] Xu L., Li W., Chen Z., Guo Q., Wang C., Santhanam R.K., Chen H. (2019). Inhibitory effect of epigallocatechin-3-O-gallate on α-glucosidase and its hypoglycemic effect via targeting PI3K/AKT signaling pathway in L6 skeletal muscle cells. Int. J. Biol. Macromol..

[29] Şöhretoğlu D., Renda G., Arroo R., Xiao J., Sari S. (2023). Advances in the natural α-glucosidase inhibitors. eFood.

[30] Terao J., Piskula M., Yao Q. (1994). Protective effect of epicatechin, epicatechin gallate, and quercetin on lipid peroxidation in phospholipid bilayers. Arch. Biochem..

[31] Shaki F., Shayeste Y., Karami M., Akbari E., Rezaei M., Ataee R. (2017). The effect of epicatechin on oxidative stress and mitochondrial damage induced by homocysteine using isolated rat hippocampus mitochondria. Pharm. Sci. Res..

[32] Cremonini E., Fraga C.G., Oteiza P.I. (2019). (–)-Epicatechin in the control of glucose homeostasis: Involvement of redox-regulated mechanisms. Free Radic. Biol. Med..

[33] Tajuddeen N., Sallau M.S., Musa A.M., Yahaya S.M., Habila J.D., Ismail A.M. (2016). A novel antimicrobial flavonoid from the stem bark of *Commiphora pedunculata* (Kotschy & Peyr.) Engl. Nat. Prod. Res..

[34] Jung E.K., Sang S.K., Chang-Gu H., Nam H.L. (2012). Antioxidant Chemical Constituents from the Stems of *Cleyera japonica* Thunberg. Int. J. Pharmacol..

[35] Abdullahi S.M., Musa A.M., Abdullahi M.I., Sani Y.M., Atiku I. (2017). Catechin from the leaf extract of *Ziziphus mucronata* Willd. (Rhamnaceae). Niger. J. Pharm. Sci..

[36] Abd El-Razek M.H. (2007). NMR Assignments of Four Catechin Epimers. Asian J. Chem..

[37] Kainsa S., Singh R. (2016). Flavan-3-ol Isomers Isolated from *Euphorbia Thymifolia* Linn. Pharmacogn. Commun..

[38] Hsu H.Y., Wen M.H. (2002). Lipopolysaccharide-mediated reactive oxygen species and signal transduction in the regulation of interleukin-1 gene expression. J. Biol. Chem..

[39] Wang H., Cao Z. (2014). Anti-inflammatory Effects of (−)-Epicatechin in Lipopolysaccharide-Stimulated Raw 264.7 Macrophages. Trop. J. Med. Res..

[40] Chun J.H., Henckel M.M., Knaub L.A., Hull S.E., Pott G.B., Ramirez D.G., Reusch J.E., Keller A.C. (2022). (−)-Epicatechin Reverses Glucose Intolerance in Rats Housed at Thermoneutrality. Planta Medica.

