# Supplementary material for: Epicatechin Isolated from Litchi chinensis Sonn. (Litchi) Fruit Peel Ethyl Acetate Extract Modulated Glucose Uptake in Chang Cells and Suppressed ROS Production in RAW 264.7 Macrophages
Source: Antioxidants (Basel). 2024 Oct 14;13(10):1233. doi: 10.3390/antiox13101233 (PMC11505627; doi:10.3390/antiox13101233)
Supplement: Supplementary file 1 [file antioxidants-13-01233-s001.zip › antioxidants-3222655-supplementary.DOC.pdf]

SUPPLEMENTARY MATERIALS

Table S1: Non-polar to polar eluent system used gradient column fractionation of the EtAc extract

Solvent system	v:v:v ratio (mL)
DCM: MEOH: WATER	9:1:0.125
DCM: MEOH: WATER	8:2:0.1875
DCM: MEOH: WATER	7:3:0.281
DCM: MEOH: WATER	6:4:0.422
DCM: MEOH: WATER	5:5:0.633
MEOH: ETHYL: WATER	5:5:0.633

Table S2: Yield of fractions from gradient column chromatography of the EtAc extract

Fraction	Yield (g)
1	0.2
2	0.12
3	0.14
4	0.51
5	0.47
6	0.81
7	0.13
8	0.11

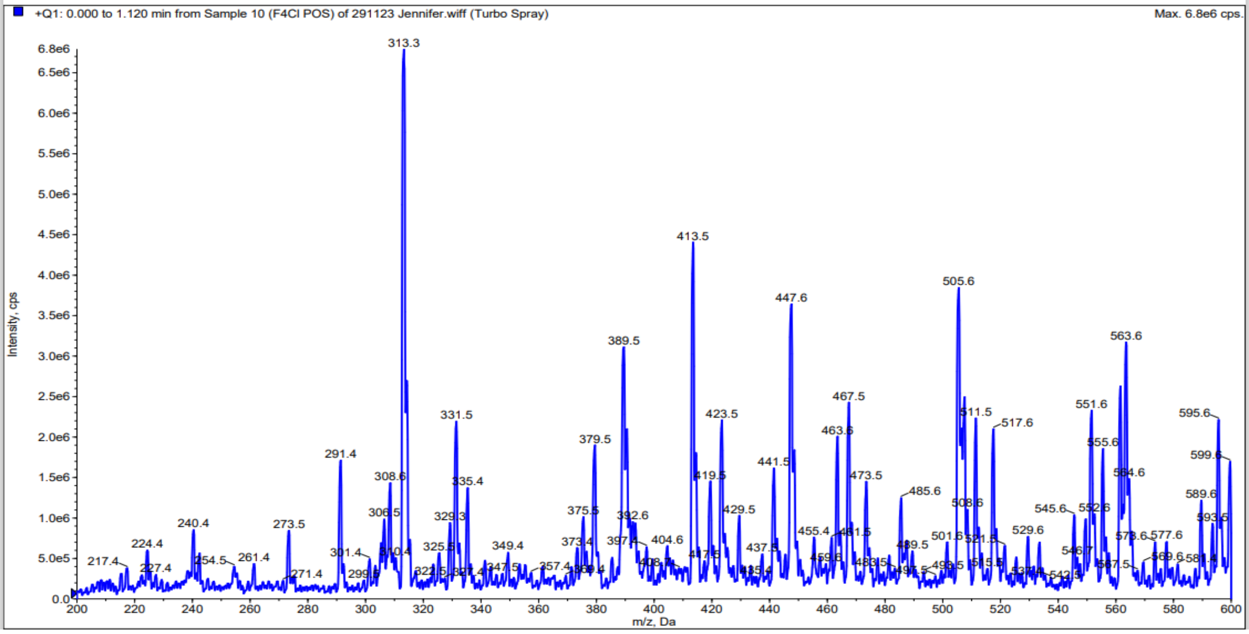

Figure S1: ESIMS (+) spectrum of the isolated compound

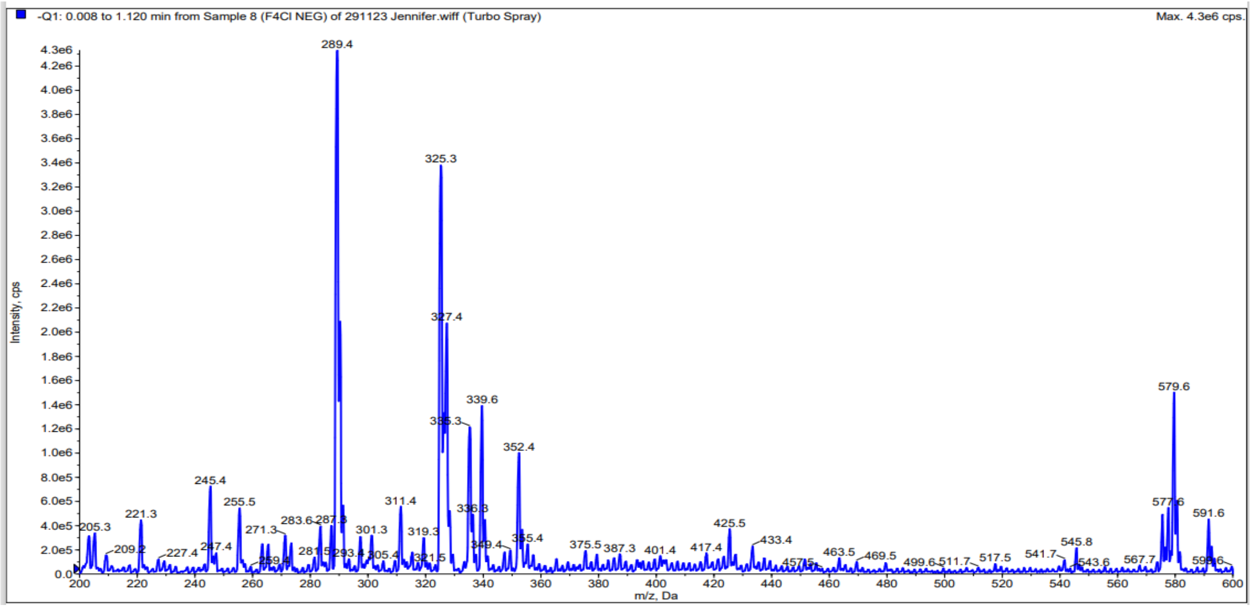

Figure S2: ESIMS (-) spectrum of the isolated compound

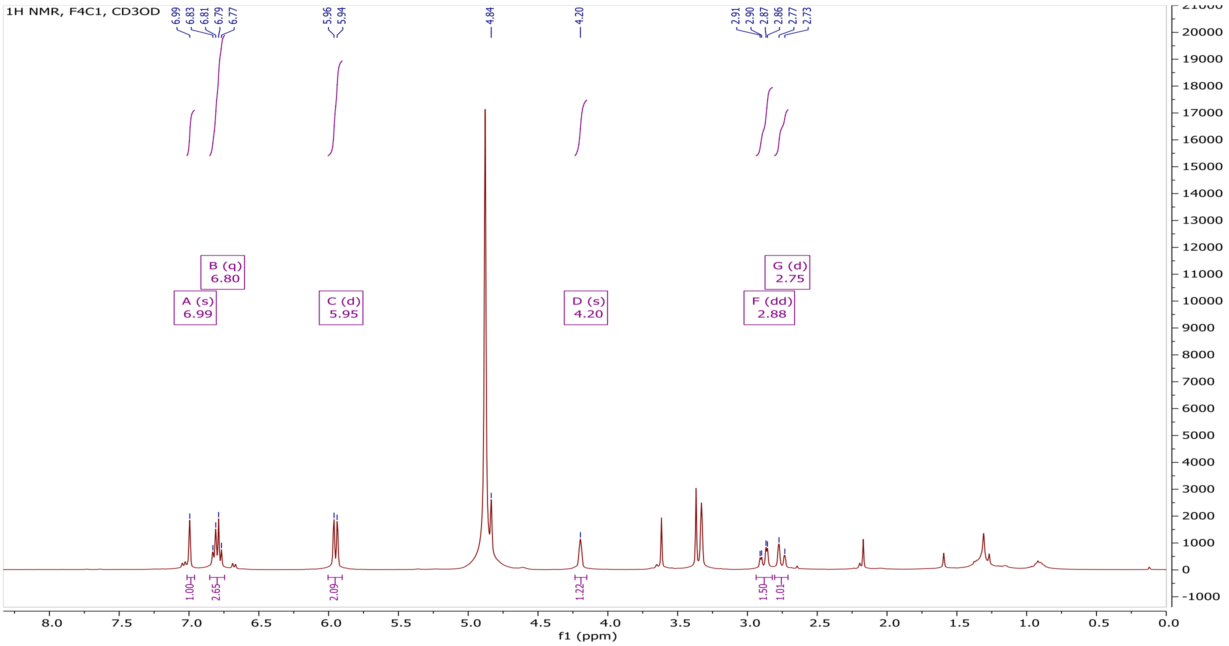

Figure S3: ^{1}H NMR (400 MHz, CD_3OD) spectrum of the isolated compound

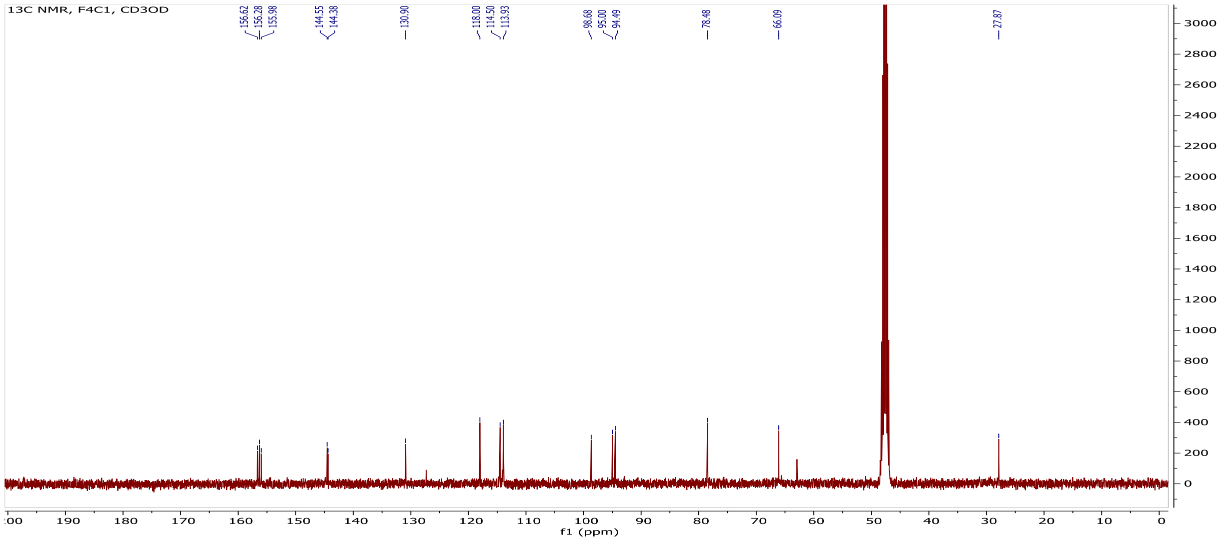

Figure S4: ^{13}C NMR (100 MHz, CD_3OD) spectrum of the isolated compound

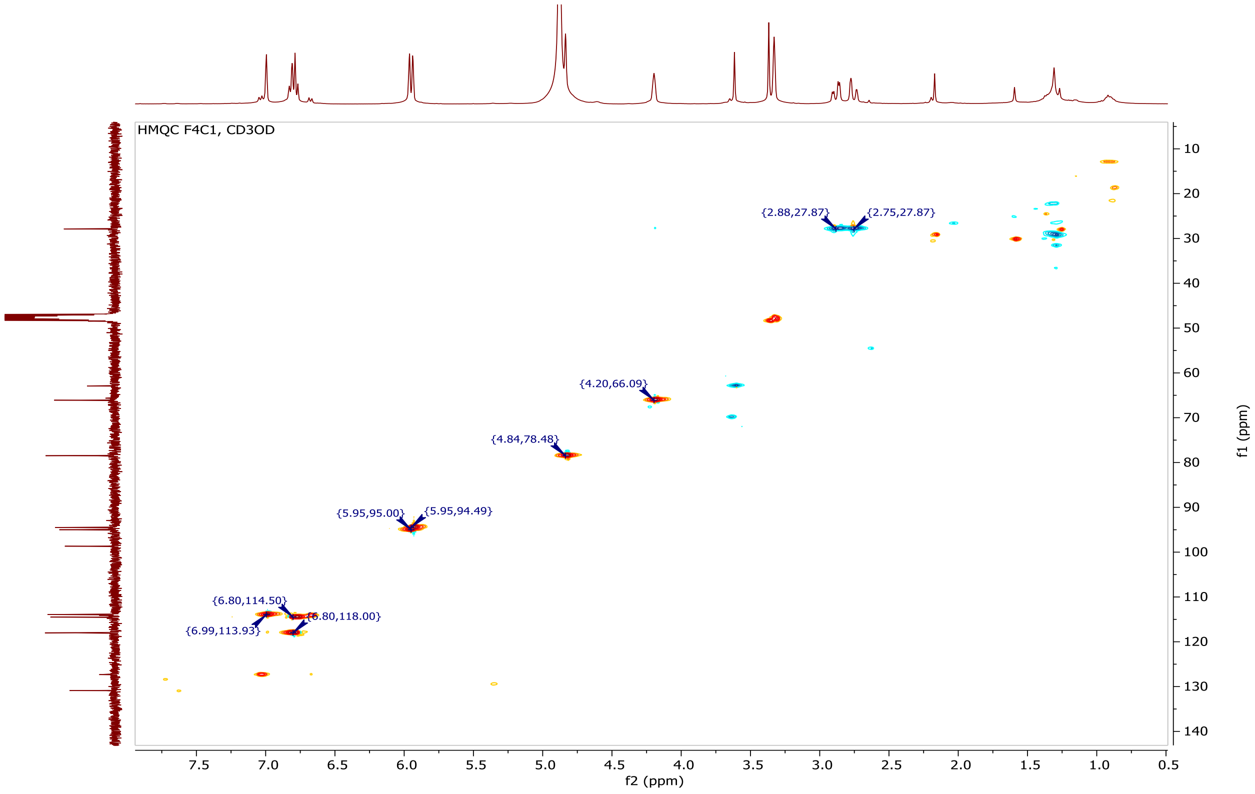

Figure S5: HSQC spectrum (CD_3OD) of the isolated compound

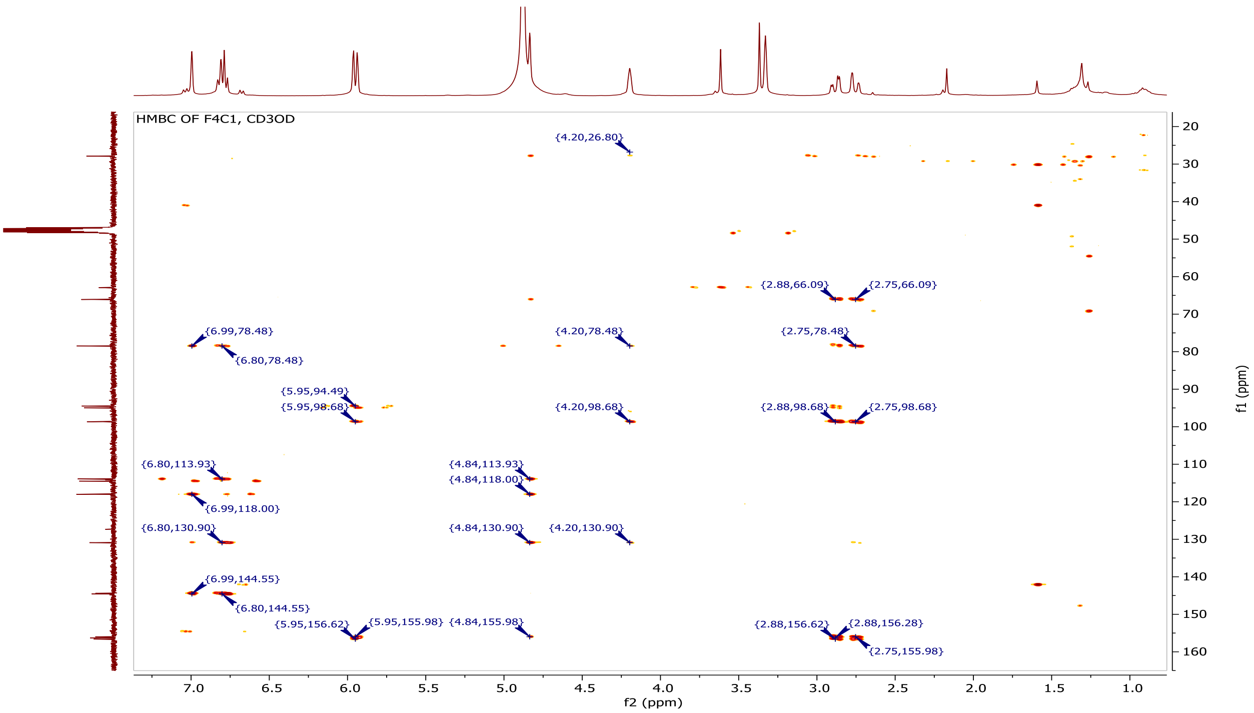

Figure S6: HMBC spectrum (CD_3OD) of the isolated compound

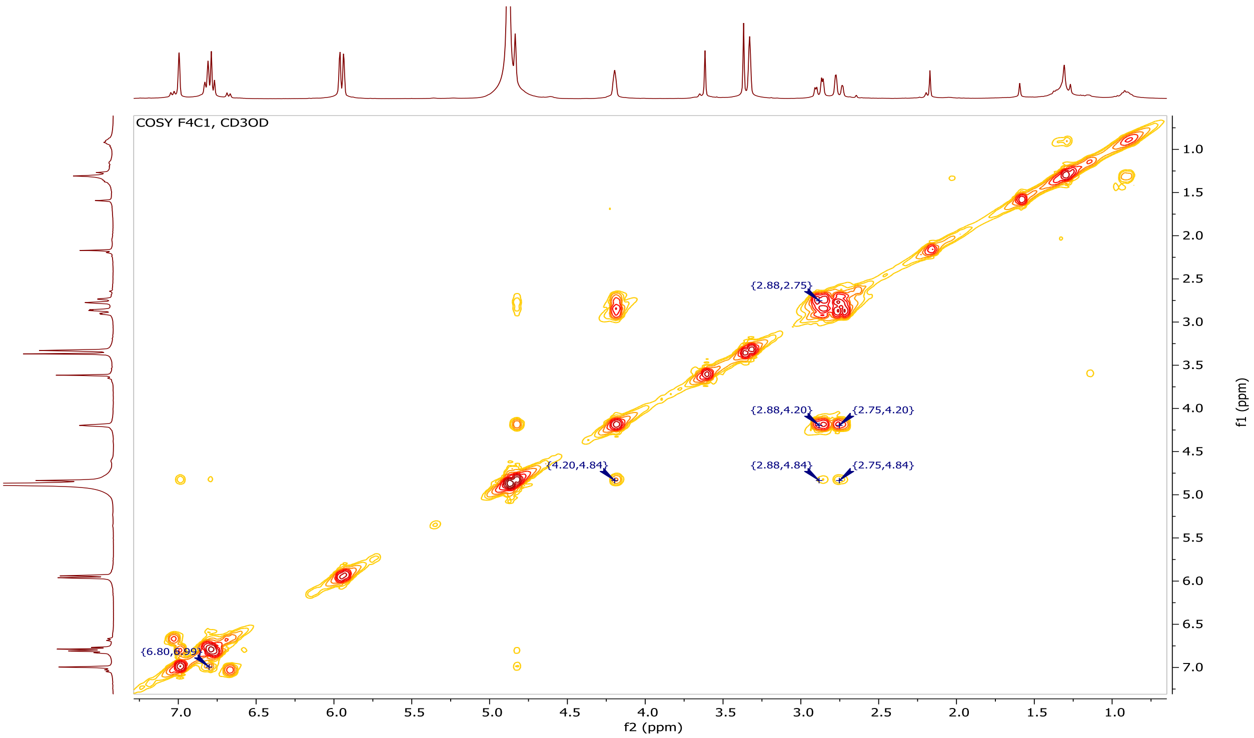

Figure S7: COSY spectrum (CD_3OD) of the isolated compound

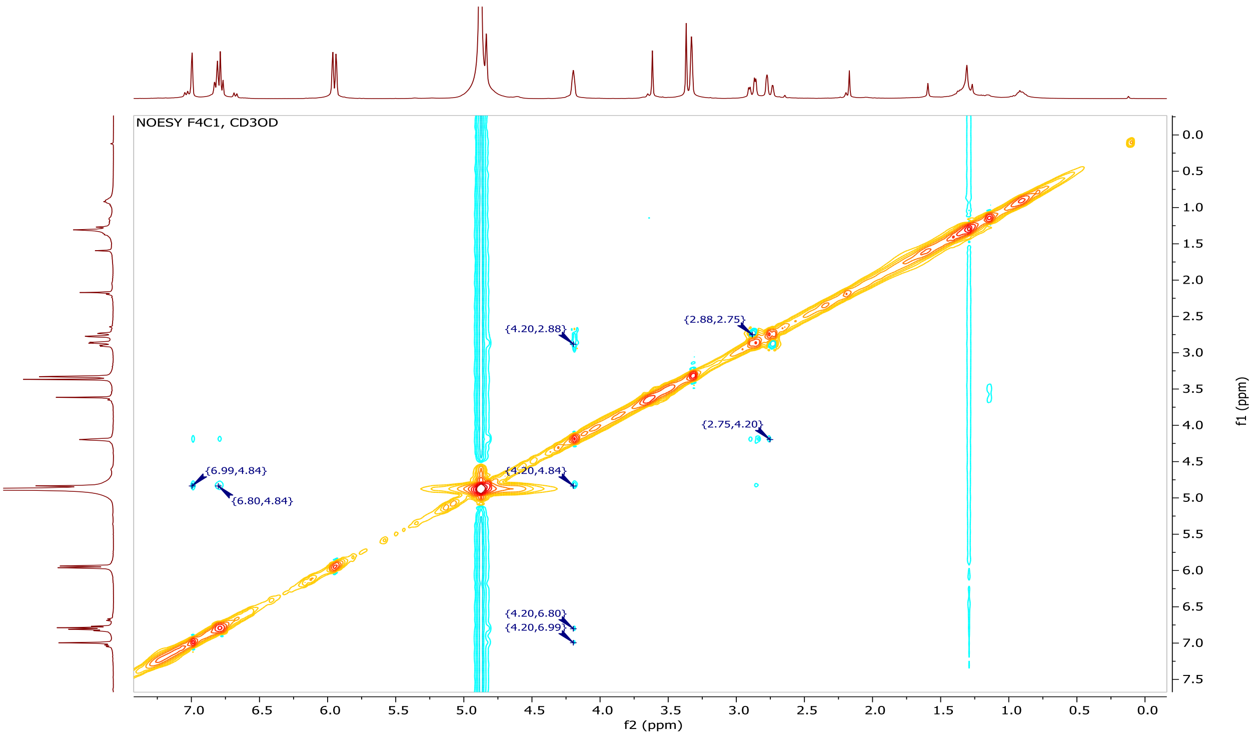

Figure S8: NOESY spectrum (CD_3OD) of the isolated compound
